# Wheat *Argonaute 5* Functions in Aphid–Plant Interaction

**DOI:** 10.3389/fpls.2020.00641

**Published:** 2020-05-26

**Authors:** Phumzile Sibisi, Eduard Venter

**Affiliations:** Department of Botany and Plant Biotechnology, University of Johannesburg, Auckland Park, South Africa

**Keywords:** ARGONAUTE protein, the Russian wheat aphid, plant defense modulation, arthropod–plant interaction, virus response

## Abstract

Aphids feeding on plants experience similar responses to pathogens due to the prolonged and intimate contact with the plant. *Diuraphis noxia* is an economically important aphid pest on wheat that exhibits such an interaction. Studies on small RNA (sRNA) that regulate genes imparting resistance to wheat against *D. noxia* have predicted an *Argonaute 5* (*TaAGO5*) gene as possible role player in the resistance response. Functional characterization revealed that *TaAGO5* is crucial in regulating the response to infestation by *D. noxia*. Knockdown of *TaAGO5* by 22% in *D. noxia* resistant wheat resulted in a completely susceptible phenotype. The fecundity and stress levels of *D. noxia* feeding on these silenced plants were similar to aphids feeding on the susceptible controls. Thus, *TaAGO5* is crucial in the defense response by wheat plants during aphid feeding and this is similar to *Nicotiana benthaminia* plants experiencing arthropod herbivory. Additionally, *TaAGO5* was differentially regulated by the Barley mosaic virus (BMV) used in the functional characterization. This provides evidence that *TaAGO5* could play a role during virus infection of wheat. The role of AGO5 proteins in plant responses to arthropod herbivory and virus infection is known for dicotyledonous plants. Here, we present data that indicate that this role of *TaAGO5* is conserved in wheat and possibly for monocotyledonous plants. These observations extend our knowledge on the roles of AGO proteins in plant resistance.

## Introduction

Concerted infestation of wheat by *Diuraphis noxia* (Kurdjumov, the Russian wheat aphid) leads to extensive economic losses if untreated. The preferred hosts of this specialized aphid are wheat, barley, related cereals, and some grasses. This contrasts it to other well studied generalist aphid pests, e.g., *Myzus persicae* and *Bemisia tabaci*. *D. noxia* only reproduces asexually through parthenogenesis in most regions of its distribution and is not effective in harboring and transmitting viruses (Cronjè, 1990). These attributes make it a useful system to study monocotyledonous–arthropod and specialized plant–arthropod interactions. With completion of its genome sequence, more tools are becoming available, even though annotation shortcomings and public availability impede progress (Nicholson et al., 2015; Burger and Botha, 2017). *D. noxia* occurs as wingless females (apterous) in affected areas, but can readily develop wings (alate) under adverse conditions. This, coupled with its rapid reproduction rate, can detrimentally affect infected fields in short periods of time and once the aphid colonizes susceptible wheat, it accrues in large numbers per plants. This level of prolonged and intensive feeding results in leaf rolling and the formation of chlorotic lesions that extend to chlorotic streaking in highly susceptible plants (du Toit and Aalsberg, 1980). Although not isolated yet, it is hypothesized that extensive effector secretion induces the different phenotypic effects when feeding (Nicolis and Venter, 2018). The aphid is unique in that it changes the turgor pressure in leaf cells to allow the leaves to roll up. This provides a protected environment for the aphids against predatory insects like wasps and makes it difficult to treat infestations with contact insecticides. Severe infestation results in lowered yield due to less or malformed seed production, head trapping with no seed production, and ultimately the death of plants. The development of chlorotic lesions and streaking is the result of chloroplast damage, possibly to create a photosynthate sink (Botha et al., 2006). Identification of effector proteins from *D. noxia* should allow for the elucidation of the pathways involved with the phenotypical responses that are induced in the infested wheat plants.

Aphids, like pathogens, have intimate relationships with host plants. During the establishment of this feeding relationship, *D. noxia*, like other aphids, will alight on the wheat plant and probe with its stylet for a suitable feeding location. This probing results in the aphid inserting its stylet between mesophyll cells. As the stylet moves inter-cellularly occasional probing into adjacent cells occur, possibly to determine its location in the leaf. Once the sieve tube is found, the aphid will start ingesting phloem and occasionally extract its stylet and insert it into the xylem to drink water (Tjallingii, 2006; Will et al., 2007). This intimate interaction with plant cells exposes the aphid to the innate immune response and the secretion of gelling saliva during probing protects against this contact. This thicker saliva forms a protective layer around the stylet and also plugs holes made into cells while the stylet travels through the leaf. Once the stylet penetrates the sieve tube, the aphid produces copious amounts of watery saliva (Tjallingii, 2006; Will et al., 2007). This functions in the establishment of a feeding site and releases effector proteins into the phloem to allow extended feeding at a site (Bos et al., 2010).

Incorporation of resistance genes (*Dn*) into wheat cultivars is the best strategy to combat *D. noxia*. There are currently ca. 14 identified *Dn*-genes that are used to varying degrees in breeding programs (Lapitan et al., 2007). To date, only *Dn 2401* was successfully identified as *Epoxide hydrolase 2* through long-read sequencing, but this still needs functional validation (Tulpová et al., 2019). However, the development of new resistance breaking *D. noxia* biotypes is necessitating the continued search for new resistance sources and this lack of knowledge on the plant resistance genes and the effector proteins from *D. noxia* hampers our understanding of the interaction. Although several studies into gene regulation in wheat infested by *D. noxia* were performed, only one study focused on the regulation and role of small RNA (sRNA) (Nicolis et al., 2017). This regulation is effected through the RNA induced silencing complex (RISC), where the miRNA pairs with the ARGONAUTE (AGO) effector protein complex that sequesters the target mRNA based on sequence similarity (Iwakawa and Tomari, 2013). The RISC can silence expression of a gene through cleavage or translational repression depending on the similarity in the miRNA:mRNA complex (Baulcombe, 2004; Voinnet, 2009).

Central to the RISC is the AGO effector proteins that are found in both prokaryotes and eukaryotes, including plants (Cerutti and Casas-Mollano, 2006). The AGO proteins contain four functional domains with the PAZ, MID and PIWI domains conserved and the N-domain displaying the most variation between different species (Tolia and Joshua-Tor, 2007; Singh et al., 2015). The PAZ and MID domains play a role in binding the 5’monophosphorylated and the 3’ nucleotides of the sRNA (Mi et al., 2008; Takeda et al., 2008). Mutation studies in *Drosophila melanogaster* indicated that the loss of the PAZ domain does not result in the loss of sRNA binding; however, shorter sRNA molecule binding is favored (Hur et al., 2013). In plants, this may differ, as the association with the sRNA is based on the interaction with the 5’monophosphorylated nucleotide (Tolia and Joshua-Tor, 2007). The PIWI domain effects the cleavage of the associated mRNA and mutation of conserved residues results in a reduction of mRNA suppression (Hur et al., 2013). Loss of the N-domain results in a constitutive activation of PIWI-based cleavage of sRNAs (Hur et al., 2013). These structural differences and their influence on regulation of gene expression hint at a complex interplay between the different AGO proteins, sRNAs, and their mRNA targets.

Limited knowledge of the wheat–*D. noxia* interaction hampers our efforts to breed more durable wheat cultivars. *D. noxia* adapts at an increasing rate with prolonged exposure to the limited number of *Dn*-genes deployed in the field. Interestingly, a new biotype may have the ability to overcome multiple resistance genes, even ones that were not deployed in the field in large numbers. This was evident when the second South African biotype (RWASA2) was detected (Jankielsohn, 2016) and it developed resistance against *Dn1*, *2*, *3*, *8*, *9*, and *Dn2006* simultaneously. These *Dn*-genes were mapped to different chromosomes in the wheat genome and they could be related genes from a similar family and the development of a new effector possibly affects all four. This implies that these genes make use of a dedicated arthropod resistance pathway that is targeted and perturbed by an effector or effectors that target components of this pathway. Indeed, the discovery of *TaAdnr1*, an integrated-domain nucleotide-binding leucine-rich repeat (NLR-ID), that functions similarly to *RRS1* of *Arabidopsis* supports the occurrence of dedicated resistance response pathways in wheat (Nicolis and Venter, 2018). Regulation of the *Dn*-genes and pathways associated with the resistance response is influenced by sRNA through the RISC. To complement our knowledge on this part of the plant–arthropod pest interaction, we have studied the role of an AGO5-like protein through functional characterization.

## Materials and Methods

### Plant Material and Aphid Infestation

Tugela (*D. noxia* South Africa biotype 1, RWASA1, susceptible near-isogenic line) and Tugela DN (RWASA1 resistant near-isogenic line carrying the *Dn1* resistance gene introgressed from PI 137739) wheat used in this study were obtained from the Agricultural Research Council-Small Grain (ARC-SG), Bethlehem, South Africa. The seeds were planted in a mixture of sterilized potting soil and compost (2:1) with pots seeded to yield five plants per pot and thinned if needed and the number of pots planted were adjusted to the specific experiment. The plants were grown under controlled conditions at 22°C with a 12 h photoperiod and watered every second day. *D. noxia* RWASA1 used for this study was obtained from the ARC-SG. The aphid colony was reared on RWASA1 susceptible wheat PAN 3434 (Pannar) under controlled conditions at 19°C with a 12 h photoperiod and watered three times a week. Plants were infested at the two leaf stage (Zadoks seedling growth stage 12) with 20 *D. noxia* RWASA1 aphids per plant.

### Analysis of *T. aestivum* ARGONAUTE 5 Protein

ARGONAUTE proteins are well known role players in the regulation of gene expression through sRNA silencing of gene transcripts. Prediction of TraesCS3B02G287600 (*TaAGO5*, July 2017 Triticum *aestivum* EnsemblePlants release) as a target of a miRNA from a concurrent study led to the hypothesis that it may play a role in the response of wheat to *D. noxia* infestation (Sibisi, 2020). The coding region of *TaAGO5* was amplified from both Tugela (RWASA1 susceptible) and Tugela DN (RWASA1 resistant) using cDNA and sequenced. These primers were designed using the sequenced genome of Chinese Spring available at Ensemble. The sequences from both cultivars were generated and aligned with Clustal Omega^1^ to TraesCS3B02G287600 to identify polymorphisms.

Differential expression of *TaAGO5* was measured using reverse-transcription quantitative PCR (RT-qPCR) at seven time points 0, 1, 4, 6, 12, 24 and 48 h post infestation (hpi). Three biological repeats were performed and the leaf material was sampled into liquid nitrogen. Total RNA was isolated separately from each leaf using Trizol reagent (Ambion) and first strand cDNA synthesis was performed with Superscript IV (ThermoFisher) according to the manufacturers’ specifications. The expression was evaluated on a Bio-Rad CFX96 Connect system. After optimization of *TaAGO5* amplification, the reactions contained 0.5 X iTaq universal SYBR Green supermix (Bio-Rad), 0.5 μM specific forward and 0.5 μM reverse primer and cDNA (1:19 dilution of the cDNA synthesis reaction) to a final volume of 10 μl. The cycling conditions were an initial denaturation for 2 min at 95°C followed by 40 cycles of amplification and quantification consisting of 15 s denaturing at 95°C, 20 s annealing at primer specific temperatures (T_M_) (Supplementary Table S1), 10 s primer extension at 72°C. The melt curve analysis consisted of a single denaturation step at 95°C followed by cooling to 65°C at 20°C per second. The fluorescence signal was collected between 65 and 95°C at 0.2°C per second. All amplicons were initially sequenced to ensure specificity and all wheat cDNA was tested for aphid RNA contamination using aphid specific primers (Chen et al., 2000). The quantification cycle values (Cq) were exported into REST software 2009 (Qiagen) (Pfaffl, 2001). Two reference genes *18S rRNA* and *GAPDH* were used as internal controls to normalize expression levels (Nicolis et al., 2017). The calibrated normalized relative quantification values were exported into Microsoft^®^ Excel 2010 where expression, standard deviation, and errors were calculated from the three biological replicates. Significantly different expression was calculated between uninfested (control) and infested samples, within resistant and susceptible cultivars and between resistant and susceptible cultivars using ANOVA (*p* < 0.05) followed by Tukey HSD.

### Virus-Induced Gene Silencing of *TaAGO5*

To perform silencing of *TaAGO5* using barley stripe mosaic virus (BSMV) VIGS, the sequence was analyzed for unique regions. The silencing insert was designed to target a 150 bp fragment of the PIWI domain that was unique to *TaAGO5* within the wheat genome. This fragment was cloned into the BSMVγ clone of the BSMV system and sequence specificity and orientation were confirmed. The VIGS experiment was performed according to Scofield et al. (2005) and the T7 mMESSAGE mMACHINE transcription system (Life Technologies) was used to generate the RNA transcripts. Viral controls included BSMV_0_, derived from the empty pSL038-1 vector, and BSMV_PDS_ that included a transcript targeting the *phytoene desaturase* gene to act as a visual marker of correct viral reconstitution. Uninoculated controls of Tugela and Tugela DN were also included.

Tugela and Tugela DN were used in the silencing of *TaAGO5*. Thirty plants of each cultivar were inoculated with the BSMV silencing vector. Inoculation of the second leaf was performed at emergence of the third leaf. Fifteen inoculated plants were infested with RWASA1 and 15 inoculated plants were uninfested serving as controls for viral inoculation. RWASA1 infested Tugela and Tugela DN plants that were not inoculated with any viral construct served as susceptible and resistant controls. Each treatment group consisted of fifteen plants grown under greenhouse conditions inside of insect cages in order to prevent early aphid infestation prior to viral inoculation. Each plant was observed as an independent biological repeat.

Aphid reproduction was measured as an indicator of the effect of silencing of *TaAGO5* has on the resistance response. Five days after viral inoculation, clip cages were placed on the emergent third leaf in 36 treated and 12 untreated plants. A single adult apterous aphid was placed inside each clip cage and the plant was infested with an additional 20 RWASA1 aphids. The following day, all aphids except one newborn nymph were removed from the clip cage and this nymph was regarded as the foundress. Nymphs born to this foundress were counted and removed every 24 h for 7 days after appearance of the first nymph. The intrinsic rate of increase (r_m_) of each foundress was estimated according to the formula r_m_ = (0.738 × ln(M_d_))/d, where M_d_ is the number of nymphs produced in a period equal to the prenymphipositional period (d) (Wyatt and White, 1977).

Five days after aphid infestation (10 days after viral inoculation), the third leaf and aphids were harvested from three experimental plants per treatment, quick frozen in liquid nitrogen, and stored at −80°C until needed. Total RNA was isolated separately from each leaf and aphids using Trizol reagent and first strand cDNA synthesis was performed with Superscript IV. RT-qPCR was used to confirm the silencing of *TaAGO5* and the regulation of *TaAGO1* (JQ805149), *TaAGO2* (KY794780), and *TaAGO4* (JQ805150) as controls to ensure that the silencing was evident only for *TaAGO5*. The RT-qPCR analyses were performed on triplicate bioreps as above. RT-qPCR data were statistically analyzed between control (untreated and uninfested) and treated uninfested and treated infested samples within the genotype as before. For *D. noxia*, gene expression levels of *Heat Shock Protein 70* (*HSP70*) were determined against validated *Actin* and *L32* as reference genes (Gerardo et al., 2010; Kang et al., 2017). This was used as a molecular marker of stress experienced by the aphids. *D. noxia* that were feeding on untreated Tugela and untreated Tugela DN were used as a control. The significance of expression was determined as before.

To measure the plant biomass all plant material was harvested at 21 days after viral inoculation, all aphids were removed from the remaining plants per treatment and the aboveground (shoot) plant biomass was separated from the roots. The roots were rinsed and together with the aboveground plant biomass dried for 48 h at 40°C and their weight determined.

### Statistical Analyses

All the experiments were performed as three biological repetitions with data collected and analyzed from all three repetitions. In the VIGS experiments, each plant was treated as a biological repeat. The data generated during the study were statistically analyzed in SPSS (IBM Corporation). Data were tested for normality using the Shapiro–Wilk test and the homogeneity of variance was tested using Levene’s test. ANOVA was used to test the effect of treatments followed by Tukey’s HSD to determine significant differences.

## Results

### Role of *Argonaute 5* in the Wheat–*D. noxia* Interaction

**FIGURE 1:**
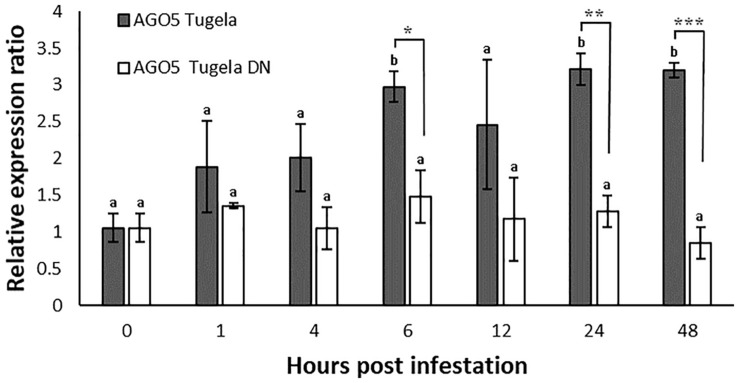
Expression analysis of *TaAGO5* in *D. noxia* susceptible Tugela and resistant Tugela DN isogenic lines. Differential expression was measured against the standardized 0 hpi as control. Values are the means of three biological repetitions with significantly different regulation indicated by *p ≤ 0.05, **p ≤ 0.01, and ***p ≤ 0.005 between cultivars. Different letters indicate time points that are significantly different to 0 hpi (*p* ≤ 0.05) as determined by ANOVA and Tukey’s HSD *post hoc* test.

Expression analysis for *TaAGO5* after aphid infestation showed no evident regulation of this gene in the resistant cultivar Tugela DN (Figure 1). In the susceptible cultivar Tugela, the gene was upregulated threefold at 6, 24, and 48 hpi compared to 0 hpi. At these time points, the gene was also significantly upregulated in Tugela compared to Tugela DN at each time point. The level of induction increased and stabilized at threefold over the comparative time points.

**FIGURE 2:**
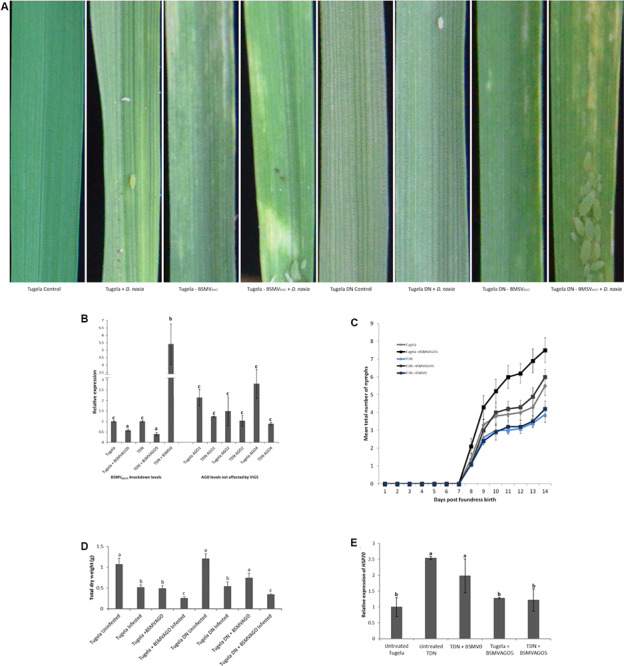
Knockdown of *TaAGO5* using Barley stripe mosaic virus (BSMV)-mediated virus-induced gene silencing. **(A)** Phenotypic variation after different treatments observed 20 days after virus infection. The controls were untreated-uninoculated plants and displayed no lesions. Untreated-infested plants served as symptom controls, with Tugela plants showing longitudinal streaking and leaf rolling in contrast to only localized cell death observed for Tugela DN. Tugela and Tugela DN BSMV_AGO_ plants displayed severe chlorotic streaking and leaf rolling characteristic of the susceptible phenotype. **(B)** Knockdown of *TaAGO5* after BSMV infection of the plants. Both Tugela and Tugela DN (TDN) *TaAGO1*, *2*, and *4* were evaluated for response to the silencing of *TaAGO5*. The Tugela DN empty vector control (BSMV_0_) showed high levels of induction. **(C)** Mean total aphid production of 10 plants per treatment over 14 days. Aphids feeding on Tugela DN displayed the lowest mean production of nymphs. In contrast, the aphid numbers were highest for Tugela DN and Tugela knockdown plants. **(D)** Total dry weight for the different plants after 14 days of feeding by *D. noxia*. Biomass was the highest in the untreated controls and lower for the infested plants. The knockdown of *TaAGO5* in Tugela DN resulted in biomass lower than the infested controls. Treated and infested plants displayed the lowest biomass. **(E)** Expression analysis of *HSP70* as measure of increased stress levels of *D. noxia* feeding on knockdown plants and controls. Increased levels were displayed on the resistant Tugela DN and the BSMV_0_ controls. The elevated levels for Tugela DN are in line with the antibiotic effect imparted by *Dn1* on the cultivar. The observed elevated levels of *HSP70* in the BSMV_0_ control indicated that the resistance response of the Tugela DN cultivar against *D. noxia* feeding was intact. Different letters indicate significant differences to the controls (*p* ≤ 0.05) as determined by ANOVA and Tukey’s HSD *post hoc* test.

Knockdown of *TaAGO5* was accomplished using VIGS with a silencing fragment designed from the PIWI domain. This fragment was screened against the wheat genome to ensure specificity and was found to be unique to the PIWI domain of *TaAGO5*. Additionally, the regulation of *TaAGO1*, *2*, and *4* was followed to ensure that only *TaAGO5* was affected by the construct. Knockdown of *TaAGO5* showed that an efficiency of 43 and 22% was reached for Tugela and Tugela DN, respectively (Figure 2B). The knockdown level for Tugela was similar to previous gene reports for this interaction (Nicolis and Venter, 2018), but the Tugela DN levels were knocked down to higher remaining levels. The selected control plants to test the effect of the empty vector in the experiment, resulted in fivefold higher levels of *TaAGO5* being induced. The *TaAGO1*, *2*, and *4* levels were not affected by the silencing of *TaAGO5* (Figure 2B). Knockdown of *TaAGO5* resulted in phenotypical changes in the treated plants exposed to aphid infestation (Figure 2A). This manifested as increased chlorosis in both Tugela and Tugela DN upon infestation and visible leaf rolling for the treated Tugela DN, which are absent in the untreated Tugela DN plants. The observed levels of chlorosis were higher than that observed in untreated Tugela controls infested with aphids. The empty vector control plants did not deviate from the untreated plants (Supplementary Figure S1). This was also observed as a lower level of plant biomass accumulation (Figure 2D) in the treated plants compared with the control and untreated Tugela DN plants upon infestation. The levels were similar to the susceptible Tugela when infested with *D. noxia*. To further measure the effect of silencing of *TaAGO5*, we measured the effect on aphid accumulation (Figure 2C), the intrinsic rate of increase of the aphids (Table 1) and the stress level experienced by the aphids (Figure 2E). The mean aphid numbers increased faster in treated Tugela compared to the infested control Tugela plants (Figure 2C). The aphids increased at a similar rate to the Tugela control plants in the treated resistant Tugela DN plants. Thus, in both knockdown lines, the aphid increase was unfettered compared with their non-treated controls. The empty vector control did not display differential increases compared with the untreated control Tugela DN. *HSP70* regulation was taken as an additional measure of the potential stress that the aphids may experience (Figure 2E). Levels were not elevated in Tugela and both the knockdown lines. However, the levels in Tugela DN and the empty vector control increased between 2- and 2.5-fold when compared with the susceptible control Tugela plants. Enhanced levels detected on aphids feeding on the resistant Tugela DN were consistent with knowledge on aphids being stressed through the antibiotic effect the *Dn1* gene impart to the cultivar. The elevated levels in aphids feeding on the BSMV_0_ line indicate that a sustained defense response against *D. noxia* was not affected by the presence of the BSMV virus vector.

**TABLE 1:** Aphid mean intrinsic rate on BSMV_AGO5_ treated and control plants.

Treatment	Mean births/day	Intrinsic rate of increase (r_m_)	Prenymphipositional period (days)
Tugela	3.75a	0.344	7
Tugela DN	2.87	0.316	7
Tugela DN +BSMV0	2.93	0.318	7
Tugela +BSMV_AGO5_	5.46	0.384	7
Tugela DN + BSMV_AGO5_	3.92a	0.349	7

*Different letters indicate statistical significance between the r_m_. The intrinsic rate of increase (r_m_) was used as a measure of aphid fecundity for the different treatments. The highest calculated rate of increase was observed in aphids feeding on susceptible Tugela + BSMV_A__GO5_ plants (0.384). The silenced Tugela DN + BSMV_AGO5_ plants had the same r_m_ as the susceptible Tugela control (0.344 and 0.349, respectively). Aphids feeding on the resistant control Tugela DN and Tugela DN + BSMV_0_ had the lowest rate of increase (0.316 and 0.318, respectively). Aphids feeding on Tugela + BSMV_AGO5_ had a significantly higher rate of increase (0.384) compared to the Tugela, Tugela DN, Tugela DN +BSMV_0_ controls, and Tugela DN +BSMV_AGO5_ (P = 0.039, 0.001, 0.001, and 0.001, respectively).*

## Discussion

The *D. noxia* resistant wheat line Tugela DN carries the *Dn1* resistance gene that imparts antibiosis against *D. noxia*. Antibiosis manifests through an impairment of the growth, development, and survival of *D. noxia* (du Toit, 1989). This is observed phenotypically in plants by localized cell death lesions, similar to that observed for the hypersensitive response in plant–pathogen interactions, and low numbers of aphids occurring on the plants. In the near isogenic line Tugela, that lacks *Dn1*, aphid numbers increase rapidly and the plant displays the development of chlorotic lesions and leaf rolling. The exact pathways that are impaired to induce these phenotypic changes are unknown, but turgor pressure is most probable affected to induce leaf rolling. Identification of genes that play a role in the regulation of these pathways will assist in dissecting the intricate interplay that concludes in a resistance response. In this study, *TaAGO5* was differentially regulated in the susceptible cultivar when compared to the resistant. We postulated that it is upregulated in the susceptible plant to increase regulation of stress related genes, or that the aphid induces this response to coerce the plant into leveraging a non-specific response similar to pests and pathogens manipulating the jasmonic acid pathway to circumvent defense responses (Zhang et al., 2017).

Knockdown of *TaAGO5* in *D. noxia* resistant Tugela DN resulted in a highly susceptible phenotype with increased aphid fitness and fecundity observed. This would suggest that *TaAGO5* plays a significant role in regulation of the wheat defense response against *D. noxia* and possibly to other insect attacks as well. This role is similar to that observed for *NaAGO8* in *Nicotiana tabacum* against *Manduca sexta*. Results from Pradhan et al. (2017) suggested this holds true for *NaAGO5*. Knockdown of *NaAGO8* directly affected phytohormone biosynthesis and the production of nicotine, diterpenoid glycosides, and phenolamides under *MYB8* transcription factor regulation. Both *MYB8* and *MYB5* transcription factors regulate the phenylpropanoid pathway (Du et al., 2012) with *TaMYB5* also identified to function in the wheat–*D. noxia* interaction (Nicolis et al., 2017). Taken together, this may point to a similar regulation of the phytohormone and secondary metabolite pathways in the wheat–*D. noxia* interaction when compared with *NaAGO8* after arthropod feeding. The extent of the role that *TaAGO5* plays in this is not clear; however, Pradhan et al. (2017), proposes that AGO5 and AGO8 proteins may have overlapping targets based on similarity in their roles during arthropod herbivory. This may hold true for TaAGO5 and TaAGO8 proteins during the wheat defense response against the intimate feeding by *D. noxia*. Regulation effected through TaAGO5 proteins may differ marginally between different wheat–aphid interactions as wheat response against *D. noxia* is affected by the incorporated resistance gene type and the aphid biotype (Botha et al., 2010; Smith et al., 2010).

Feeding by *D. noxia* on susceptible and resistant wheat induced similar responses in *TaAGO5* regulation for susceptible plants, where a twofold induction was observed 1 h after the aphids were placed on the plants. This increased steadily and peaked at threefold induction at 48 h. Similarly, *NaAGO5* was differentially regulated in *N. tabacum* by *M. sexta* larvae oral secretions (Pradhan et al., 2017). Oral secretions elicited a six- and threefold increase in *NaAGO5* and *8* at 18 h. This differential regulation in response to *M. sexta* oral secretions was only observed for *NaAGO5* and *8*. In our study, aphids were allowed to feed freely and this may explain the lower fold expression changes observed when compared to *NaAGO5*. The complex interplay between the wheat plant and *D. noxia* feeding will affect both the aphids and the plant as the feeding progresses and aphids modulate their saliva composition and secrete effector proteins into plants to address the plant’s feeding responses (Bos et al., 2010; Mugford et al., 2016). Thus, the plant response over time will be affected by the aphid. This is not the case when exposing plants to saliva for extended durations where the plant will continue to respond only to the presence of the initial composition of the salivary proteins and not to the ever changing barrage of effectors and salivary proteins produced by the aphid in response to the plant. Due to the complexities of studying plant–arthropod interactions, the majority of aphid salivary studies are performed in artificial diets or focus on single proteins and their effects on the plant and this does not allow for the elucidation of aphid salivary modulation during prolonged feeding. How this *D. noxia* salivary modulation affect wheat plants is not clear.

*TaAGO5* is upregulated in the untreated Tugela DN and the empty VIGS vector control for Tugela DN, but not in the susceptible Tugela line when tested at 5 days after infestation. AGO proteins interact with virus and viroid sRNAs. RISCs that include AGO1, 2, 3, 4, 5, and 9 can bind to virus sRNA and *Arabidopsis* AGO5 can bind potato virus X (PVX) sRNA in *N. tabacum* and suppress the infection thereof, but only in the presence of an intact AGO2 interaction (Brosseau and Moffett, 2015). This proposes a virus response for *TaAGO5*, but only in the resistant wheat line. Infestation with *D. noxia* decreased this expression but not significantly. The upregulation of *TaAGO5* in Tugela DN occurs at a later stage during the infestation as there was no regulation detected during the time trial that spanned the initial 48 h (Figure 1). The knockdown levels of AGO proteins were only tested at 5 days post infestation and 10 days after virus inoculation. These results suggest that *TaAGO5* is expressed early on in *D. noxia* infested susceptible wheat and later in virus response in resistant wheat. These early expression levels are concomitant with that seen for the virus infection in the resistant line and the levels peak for both susceptible and resistant lines at threefold above the control.

The role of AGO5 proteins in viral defense is closely linked to AGO2 proteins. Brosseau and Moffett (2015) proposed that AGO2 proteins function in the virus infected leaf and once the virus overcomes this response, the AGO5 protein is induced systemically. This may be why *TaAGO5* regulation in response to virus infection was only observed in Tugela DN. *TaAGO2* regulation was not detected under the experimental conditions used in this study. This is most probably due to the testing of the third leaf of the wheat plants. The inoculation with BSMV was performed on the second leaf at the emergence of the third leaf and thus only virus particles that moved into the third leaf will induce this systemic effect. The difference between the susceptible and resistance line may lie in more free movement of the virus within the susceptible plant, with underlying resistance restraining this movement in the resistant plant. This may also lead to the lowered levels of knockdown observed for *TaAGO5* in Tugela DN. This lower perturbation of *TaAGO5* with a drastic phenotypical response hints at an important role for *TaAGO5* in underlying resistance responses, at minimum against viruses and aphids, but possibly in more interactions with arthropods and other viruses. AGO2 and 5 proteins were shown to act non-redundantly but additively against viruses and it was proposed that AGO5 is a follow on systemic response of AGO2 (Brosseau and Moffett, 2015). In the absence of detection of increased levels of *TaAGO2* in our experiments, we can speculate that TaAGO5 and TaAGO2 proteins will also act additively in this interaction.

## Conclusion

We postulated that *TaAGO5* is upregulated by the susceptible plant to regulate more stress related genes. Alternatively, that *D. noxia* induces this response to coerce the plant into leveraging a non-specific response, similar to pests and pathogens manipulating the jasmonic acid pathway to circumvent defense responses. Thus, increased *TaAGO5* expression in the susceptible Tugela cultivar may perturb phytohormone production and improve *D. noxia* performance in this cultivar, while this is not evident in resistant Tugela DN. The regulation of *TaAGO5* in the empty virus vector control provides evidence that this gene plays a role in viral response that is similar to that observed in dicotyledonous plants. It would be interesting to study this in other aphid interactions, where viruses are vectored by aphids.

## Data Availability Statement

The original contributions presented in the study are included in the article/Supplementary Material. Further inquiries can be directed to the corresponding author.

## Author Contributions

PS and EV designed the experiments and interpreted results. PS performed the research. Both authors co-wrote the manuscript.

## Conflict of Interest

The authors declare that the research was conducted in the absence of any commercial or financial relationships that could be construed as a potential conflict of interest.

## Footnotes

1 http://www.clustal.org/omega/
