# Supplementary material for: Wheat Argonaute 5 Functions in Aphid–Plant Interaction
Source: Front Plant Sci. 2020 May 26;11:641. doi: 10.3389/fpls.2020.00641 (PMC7266077; doi:10.3389/fpls.2020.00641)
Supplement: Supplementary file 1 [file Table_1.docx]

**Table S1**: Sequences of the primers used in this study.

| **miRNA** | **Primer type** | **Sequence** | **TM** $\boldsymbol{℃}$ | **Tm (ºC)** |
| --- | --- | --- | --- | --- |
| *GAPDH* | Forward | 5’–GCC AGT TAC CGT CTT TGG CGT C–3’ | 52 | 52 |
|  | Reverse | 5’–GGC CTT GTC CTT GTC AGT GAA G–3’ | 52 | 52 |
| *18S* | Forward | 5’–AAC ACT TCA CCG GAC CAT TCA–3’ | 52 | 52 |
|  | Reverse | 5’–CGT CCC TGC CCT TTG TAC AC–3’ | 52 | 52 |
| OligoDT | cDNA synthesis | TTTTTTTTTTTTTTTTTTTTN |  |  |
| AGO5_full length | Forward  Reverse | CATGATCGCAATGAGATTGG  GACCTGCTCAACCAGTAAAACC | 62 |  |
| AGO5_inner primer set 1 | Forward  Reverse | GATTACTCCTGTCCCCACGA  CTCACGTTCATTCGCACTGT |  |  |
| AGO5_inner primer 2 | Forward | ATCTATTGCTGCGGTGGTG |  |  |
| AGO5_VIGS | Forward  Reverse | TCGAAGACCTCTTCAGTGTCA  TCTTGCGGAAGGCAATAAGT |  |  |
| BSMV$\gamma$ | Forward | TGATGATTCTTCTTCCGTTGC |  |  |
| AGO1 | Forward  Reverse | GTGACCATAACGCCAGAGGT  CATCTTGCAGAGCGATTTCA | 52 |  |
| AGO2 | Forward  Reverse | CGCTGTGAGGTGATCAAGAA  ATGTCTGCAAGCTCCTCGTT | 52 |  |
| AGO4 | Forward  Reverse | GCTGAAGGCATGTGGAATTT  CCATCTGTCCACCACACAAG | 52 |  |
| *Actin* | Forward  Reverse | TGCCTGATGGTCAAGTCATC  TCAAGGAATGCTTTGAGCTG | 60.9 |  |
| *Heat shock protein 70* | Forward  Reverse | TCTTTCCTCATCCACCCAAG  CTCCAACCAACACAATGTCG | 52 |  |
| *Diuraphis noxia* L32 | Forward  Reverse | CGTCTTCGGACTCTGTTGTCAA  CAAAGTGATCGTTATGACAAACTCAA | 57.8 |  |
| Aphid cDNA | Forward  Reverse | TTTCCGATTAATTGAAGTAG  ATTCCTGGTCGGTTTATAAA | 52 |  |
