# Supplementary material for: Wheat Argonaute 5 Functions in Aphid–Plant Interaction
Source: Front Plant Sci. 2020 May 26;11:641. doi: 10.3389/fpls.2020.00641 (PMC7266077; doi:10.3389/fpls.2020.00641)
Supplement: Supplementary file 2 [file Image_1.pdf]

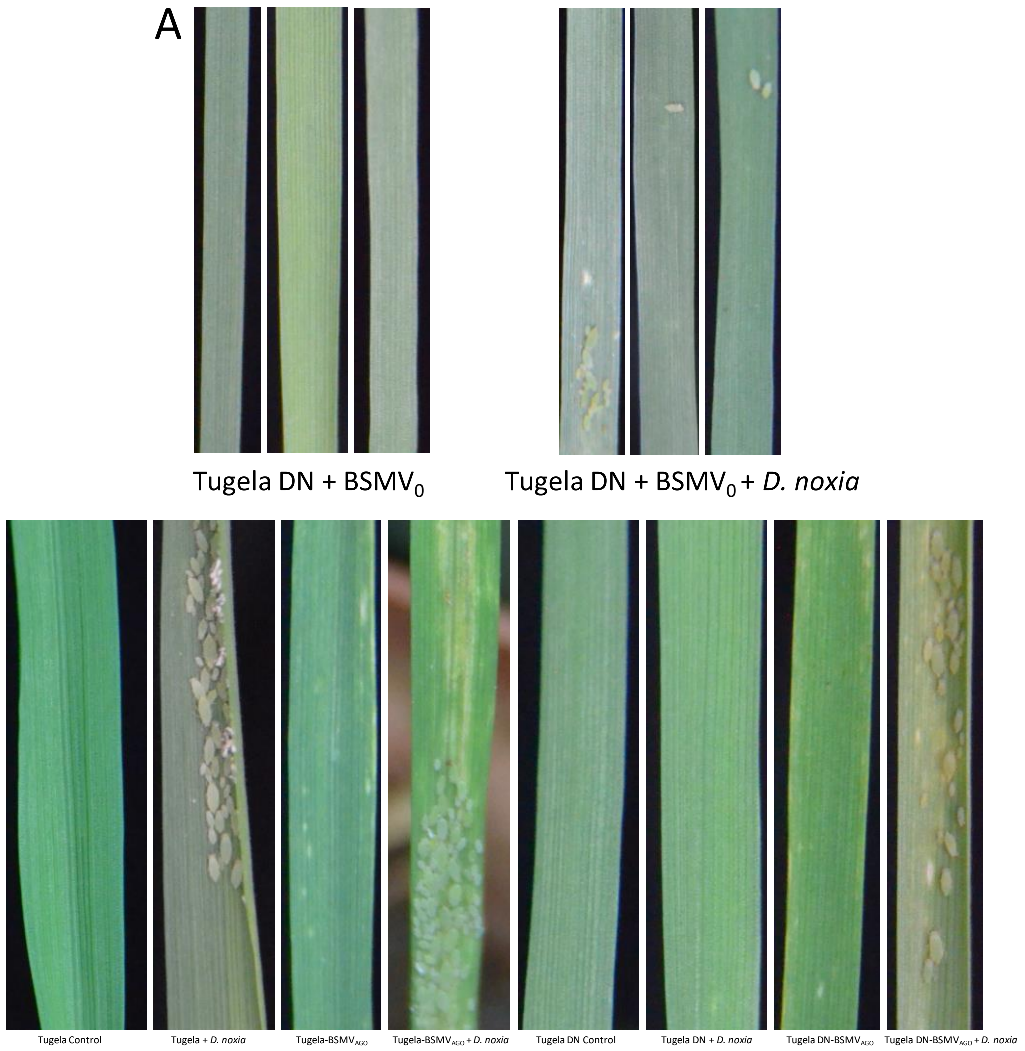

Figure S1: A) VIGS with the empty vector control. No phenotypic deviation was observed after Tugela DN plants were infected with the empty virus vector as treatment control. Phenotypic characteristics were similar to the untreated infested plants. The high levels of TaAGO5 expression in response to $BSMV_0$ does not equate to a measurable phenotypic effect. B) Second set of plants from experiments performed for Figure 1. Similar phenotypes were observed for all the plants as corresponding to those shown in Figure 1.
